# Medicine of the Chinese

**Published:** 1898-03

**Authors:** Franklin Staples

**Affiliations:** Winona, Minn.


### MEDICINE OF THE CHINESE.

BY FRANKLIN STAPLES, M. D., WINONA, MINN.

Whatever has pertained to the civilization of the Chinese has been characterized by a fixedness not known in the history of any other people. Dr. Bass aptly likens the Mongolians, in this their dominant characteristic, to what exists in the inorganic world, which, once crystalized, remains forever unchanged, with no inherent tendency either to grow or decay; while the civilization of other peoples, those of Indo-Germanic origin, he compares to what is in the organic kingdom, whose existence involves the certainty of changes incident to life. "With the latter, civilizations have sprung up, developed, bloomed and decayed, and finally perished, sometimes together with the peoples themselves." The possibility of such permanence in the habits and in what pertains to the lives of the Chinese is accounted for, in part at least, by the fact that it has ever been policy of the government of this vast empire to keep itself intellectually and physically free from intermixing with foreign races.

The material constituting the ancient history of different peoples have been transmitted to modern times in different ways. Evidences of the character and culture of the ancient Egyptians appear on remaining monuments in ancient ruins and tombs; the learning of the Greeks has come to us in the ancient Greek literature which has been preserved. Neither monuments nor historical literature are found in China to furnish history much older than the present era. Unreliable and exaggerated traditions are found, which attribute certain scientific works to persons living in the remote past. As illustrative of these, it was given that the Emperor Chin-nung (B. C. 2699), who was the reputed author of a work on medicinal herbs, had discovered in one day no less than seventy different species of poisonous plants, and at the same time others that were antidotes for the poisons of the first seventy. What belonged to ancient Sin, Chin, Sinæ, China or Seres, or to mediæval Cathay, has come down in living generations of many centuries, and exists with little change of form and substance among the Chinese of modern times. In quite recent times some innovations have been made by foreigners, and and natives educated abroad have carried the results of their learning to home institutions; but for the most part the present tense may be used in describing customs which, so far as known, have always existed.

Something is known of a Chinese literature which is extensive, and is believed by some to have had its beginning as early as the sixth century. In the ninth century the Chinese invented printing by means of engraved blocks. With these, used as stamps, fine printing was done on silk and other textile material and on paper.* Movable type made of clay is mentioned as used by the Chinese from the middle of the eleventh century. In the British Museum is a Corean book printed with movable type in 1337. The Coreans are said to have printed by means of copper type at the beginning of the fifteenth century.

### EARLY MEDICAL WORKS AND AUTHORS.

The name of Hwang-te appears as that of a Chinese emperor (B. C. 2637), who was given as the author of a treatise on medicine. There is much uncertainty concerning the time of the origin of this work, and its authorship has been doubted, in the belief that it is probably a forgery of about the beginning of the present era.† This medical work, which

*James Freeman Clark, in "Confucius and the Chinese."

‡Baas' History of Medicine. Handerson. P. 51

is entitled "Nuy-kin" or "Neiszin," is still extant; and the fact that it still serves as a medical guide is noticed as evidence of the unchangeableness and lack of progress in medicine, as in all things pertaining to human life and affairs in China.

Before the time of Hwang-te appears the mythical characacter, Chin-Nung, who is given as the good emperor who invented agriculture. The tradition is that he tested all the drugs upon his own person, before allowing them to be used upon others, and that he succeeded in prolonging his own life and the lives of his people by introducing healthful articles of food.

Che-Hwang-te (B. C. 213), who was a powerful emperor, the builder of the great Chinese wall, is noted as the burner of books. This he did in opposition to the schoolmen of the nation, who opposed him in his administration of the government. But the writings of Hwang-te, and perhaps others, escaped destruction at this time.

We have some account of the following as among early Chinese works and authors: Nang-King wrote on medicine in the second century. Wang-Shu in the third century wrote ten large volumes on the pulse. Nothing further is noted until A. D. 1247, when Sung-Tse is accredited with a work on forensic medicine. This work is said to contain valuable observations on the symptoms of drowning; and the fame of its mysterious wisdom is so great that the very sight of it is said to be enough to make poisoners, etc., confess their crimes. About A. D. 1500 appeared the Chinese cyclopædia of medicine, edited by Prince Chu-Su, of the Ming dynasty, and comprising 160 volumes, 770 treatises and 22,000 prescriptions. It was mainly from this that a committee of 800 physicians, under the presidency of Li-Shi-Chin, compiled in 1596 the fanous Pun-Tsaou-Kang-mu, or Chinese materia medica, in fifty-two volumes, describing 1890 drugs. Medical literature then degenerated for a time into shorter monographs, of which only that on acupuncture (seven volumes with copious illustrations) may be noticed. In 1740 appeared a work of ninety volumes on the pulse, with a short notice of the circulation of air in the body and the treatment of fractures; and about the same time the Pentasco, or chief Chinese work on botany was published (Withington).

A LACK OF FOUNDATION.

It has been observed that, while the history of medicine since the time of Hippocrates shows that among the poeple of the advancing nations of the world, the effort has been to establish the science of medicine upon the real foundation of anatomy and physiology, yet among the Chinese and other kindred people, such a basis for the science of medicine has been mostly unknown. With no foundation for medicine other than the demon theory of disease which has obtained among the Mongolians to the present time, no development of science has been possible. As said by Dunglison: "A variety of insurmountable obstacles have opposed themselves to the Chinese ever attaining the same degree of civilization that the European arrives at with so much comparative facility. The first is situated in his organization, whether natural or acquired by education; the second, in the frightful despotism which hangs over his head; the third, in the foolish vanity which has induced him to believe that China is the country of wisdom and the sciences."*

The religion of a people never fails to have a great part in the formation of its civilization, and in affecting its advancement in science and art. Kung-foo-tseu (Latin, Confucius), born in China (B. C. 551), became a great philosopher. He was a man of great virtue and gained an influence over the whole Chinese people. The great endeavor of Confucius was to remedy the political and moral evils of his time. He had many disciples, who recorded the sayings and maxims of their master, and the sacred books of the Chinese have preserved these precepts for the benefit of the people through all the centuries to the present time. He was successful in causing reforms, and no name stands above that of Confucius in the nation's annals. Confucianism has been called the chief religion of the Chinese. It was more an education in what pertained to material life, and in many things tended to elevate the people; but the great reformer hoped for more than has been realized in the progress of his people. Their religion has been an agnosticism, and an adherence to the worship of ancestors. Shamanism and Taoism, terms applied to Chinese religion, are but other names for sorcery. Taoism is a religion of great antiquity. It involves an implicit faith in sorcery. The Chinese have degenerated Buddhism, the religion originally an Indian product, into these religions, which have continued to the present time.† In the mythology of the Chinese, as in that of ancient Egypt and Greece, distinguished physicians are made to appear as deities; but in China such a distinction seems to be allowed principally to emperors and high officials in government. The Emperor Fuh-Hi is mentioned as the first physician and the deity of doctors. Kuang Tai Uong is the God of surgery. Ling Na is the goddess of midwifery and children. If children are sick, Taoist priests are employed in her temples to perform a ceremony for their cure. Ioh Nong Cha Su is the God of medicine and drugs. Druggists rather than physicians are his worshipers.†

---

*History of Medicine, Dunglison, p. 71.

†Berdoe, from Prof. Teile, in art. "Religions," Enc. Brit.

The veneration for ancestors and the value put upon the body after death, lead the Chinese to take great pains in the care and burial of their dead. The motive here and the object in view which prompts this care and regard for the dead, differs from that which in ancient Egypt caused the careful preservation of the dead body. The custom there resulted mainly from the belief that the same body was to be the future tenement of the soul. The time of mourning for a parent in China is three years, and for other relations in proportion. No expense is spared "in rendering the dead comfortable." "Every good Chinaman regularly every day burns incense before the tablet to his father's memory. There is in every respectable house the hall of ancestors, where the pedigree of the family, with the grandsire at the head, is inscribed, and here their descendants repair in spring to perform their devotions; then they go to the graves and present rich offerings of all kind of victuals, candies, flowers and incense; of which, however, they afterwards scruple not to make use themselves. The sums expended are often enormous"; but every one considers it his sacred duty, and no one murmurs. At stated times, when the body has mouldered into dust, they go and wash the bones and place them in an urn, which is generally preserved above ground.*

The element of superstition which appears in most things pertaining to Chinese life is illustrated by the following: The belief prevails that the infliction of demons on sufferers is by act of the gods, as punishment for sins committed as well in a supposed previous existence as in the present life. The following case among others is given by Berode: Archdeacon Grey found a grievously afflicted monk in a monastery in the White Cloud mountains. He desired to take him to the Canton Medical Missionary Hospital; but the abbot took him aside and begged him not to do so, as the sufferer had doubtless in a former state of existence been guilty of some heinous crime, for which the gods were then making him pay the well-merited penalty.†

WITHOUT GOVERNMENT CONTROL.

Concerning the governmental control, or rather the want of it in the practice of medicine in China, it is observed that the ancient and unlimited liberty of engaging in this business has not only rendered any educational standard for admission impossible, but has made the number of practitioners enormous. The following, however, as given by a German writer, while it suggests governmental interference, has a more important bearing upon what is the character of the profession. "The doctors," so runs the edict of 1882, "have the bad habit of

*From Doolittle's Social Life of the Chinese.
*Karl F. A. Gutslaff, from "China Opened."
†From "Doctoring in China," National Review, May, 1889.

not visiting their patients before one o'clock in the afternoon. Some of them even smoke opium and drink tea until late in the evening. These are abuses which the government will under no circumstances permit. Doctors must visit their patients at all times; if necessary, they must visit them several times a day. They must think more about them and less about their fees. The public and all officials are notified that a physician who does not come at once when called, can claim only half of his fees and expenses. If you physicians put off your calls, you manifest your godlessness and sin against yourselves."

### THE OUTLOOK.

Had it not been for the exclusiveness of the Chinese from all other peoples in the long past, not only more might have been known concerning what science and art may have existed among them at any time, but it is possible that more might have been added to the credit side of their account. It is known that the Chinese have long had some knowledge of the circulation of the blood, although their anatomy of the circulation is very imperfect. It is said that the Chinese inoculated for smallpox in the ninth century; yet it is known that they have goddesses of smallpox and measles that are extremely popular divinities. "Should it thunder after the pustules of smallpox have appeared, a drum is beaten to prevent them breaking. On the fourteenth day ceremonies are performed before the goddess, to induce her to cause the pustules to dry up."*

Acupuncture is largely practiced, and this is supplemented by the use of the hot iron. Many varieties of the pulse are given, each having its own significance. The wily physician will impress his patient by sitting an hour with his fingers moving in a rythmic way over the region of the pulsating artery, and then pretend to make his diagnosis and prognosis from what he has discovered. But this practice is as good and worthy as some procedures accepted and allowed in the light of English and American civilization of the present time, where persons who ought to be better educated have been known to send a lock of hair to a distant quack, or to submit to the laying on of hands and other so-called "faith-cure" performances.

A recent writer (Dr. Park) sums up concerning Chinese medicine and surgery as follows: "It is related that one of the ancient Chinese emperors directed the dead bodies of criminals to be opened; but this is questionable, since it is certain that they have the most profound ignorance of rudimentary anatomy, and glaring errors abound in their system. Being thus replete with errors, and possessing no anatomical knowl-

*Berdoe, from Doolittle's Social Life of the Chinese.

edge, their surgery was of the most barbarous type. No one dared attempt a bloody operation; the reduction of hernia was unknown; a cataract was regarded as beyond their resources, and even venesection was never practiced. On the other hand, they employed cups, and acupuncture, fomentations, plasters of all kinds, lotions and baths. The moxa, or red-hot button was in constant use, and they had their magnetizers, who appear to have been convulsionists. For a long time there existed at Pekin an Imperial School of Medicine; but now there is no such organization, nor any regulation for the privilege of practicing medicine or surgery since 1792. At least until lately the country and the cities were infested with quacks, who dealt out poison and death with impunity. They practiced most murderous methods in the place of the principles of midwifery. Only since the civilized missionaries have penetrated their country has there been any improvement in this condition of affairs."

"China opened" is an expression only recently true in any way. Native Chinese students of both sexes are now in different educational institutions in this and other countries. An educated Chinese lady having received her medical degree from the Woman's Medical College of Philadelphia, has recently become the physician to a high official in the Chinese home government. How rapidly the education of native teachers in foreign institutions, the building of hospitals, the work of missionary teachers and the extension of railroads through the great interior of China, will elevate the general standard of scientific medicine and practice, is a question commanding attention at the present time.—*Northwestern Lancet.*

